# Academic Stress and Mental Well-Being in College Students: Correlations, Affected Groups, and COVID-19

**DOI:** 10.3389/fpsyg.2022.886344

**Published:** 2022-05-23

**Authors:** Georgia Barbayannis, Mahindra Bandari, Xiang Zheng, Humberto Baquerizo, Keith W. Pecor, Xue Ming

**Affiliations:** ^1^Department of Neurology, Rutgers New Jersey Medical School, Newark, NJ, United States; ^2^Rutgers New Jersey Medical School, Newark, NJ, United States; ^3^Office for Diversity and Community Engagement, Rutgers New Jersey Medical School, Newark, NJ, United States; ^4^Department of Biology, The College of New Jersey, Ewing, NJ, United States

**Keywords:** academic stress, well-being, college students, Perception of Academic Stress, Short Warwick-Edinburgh Mental Well-Being Scale, COVID-19

## Abstract

Academic stress may be the single most dominant stress factor that affects the mental well-being of college students. Some groups of students may experience more stress than others, and the coronavirus disease 19 (COVID-19) pandemic could further complicate the stress response. We surveyed 843 college students and evaluated whether academic stress levels affected their mental health, and if so, whether there were specific vulnerable groups by gender, race/ethnicity, year of study, and reaction to the pandemic. Using a combination of scores from the Perception of Academic Stress Scale (PAS) and the Short Warwick-Edinburgh Mental Well-Being Scale (SWEMWBS), we found a significant correlation between worse academic stress and poor mental well-being in all the students, who also reported an exacerbation of stress in response to the pandemic. In addition, SWEMWBS scores revealed the lowest mental health and highest academic stress in non-binary individuals, and the opposite trend was observed for both the measures in men. Furthermore, women and non-binary students reported higher academic stress than men, as indicated by PAS scores. The same pattern held as a reaction to COVID-19-related stress. PAS scores and responses to the pandemic varied by the year of study, but no obvious patterns emerged. These results indicate that academic stress in college is significantly correlated to psychological well-being in the students who responded to this survey. In addition, some groups of college students are more affected by stress than others, and additional resources and support should be provided to them.

## Introduction

Late adolescence and emerging adulthood are transitional periods marked by major physiological and psychological changes, including elevated stress (Hogan and Astone, 1986; Arnett, 2000; Shanahan, 2000; Spear, 2000; Scales et al., 2015; Romeo et al., 2016; Barbayannis et al., 2017; Chiang et al., 2019; Lally and Valentine-French, 2019; Matud et al., 2020). This pattern is particularly true for college students. According to a 2015 American College Health Association-National College Health Assessment survey, three in four college students self-reported feeling stressed, while one in five college students reported stress-related suicidal ideation (Liu, C. H., et al., 2019; American Psychological Association, 2020). Studies show that a stressor experienced in college may serve as a predictor of mental health diagnoses (Pedrelli et al., 2015; Liu, C. H., et al., 2019; Karyotaki et al., 2020). Indeed, many mental health disorders, including depression, anxiety, and substance abuse disorder, begin during this period (Blanco et al., 2008; Pedrelli et al., 2015; Saleh et al., 2017; Reddy et al., 2018; Liu, C. H., et al., 2019).

Stress experienced by college students is multi-factorial and can be attributed to a variety of contributing factors (Reddy et al., 2018; Karyotaki et al., 2020). A growing body of evidence suggests that academic-related stress plays a significant role in college (Misra and McKean, 2000; Dusselier et al., 2005; Elias et al., 2011; Bedewy and Gabriel, 2015; Hj Ramli et al., 2018; Reddy et al., 2018; Pascoe et al., 2020). For instance, as many as 87% of college students surveyed across the United States cited education as their primary source of stress (American Psychological Association, 2020). College students are exposed to novel academic stressors, such as an extensive academic course load, substantial studying, time management, classroom competition, financial concerns, familial pressures, and adapting to a new environment (Misra and Castillo, 2004; Byrd and McKinney, 2012; Ekpenyong et al., 2013; Bedewy and Gabriel, 2015; Ketchen Lipson et al., 2015; Pedrelli et al., 2015; Reddy et al., 2018; Liu, C. H., et al., 2019; Freire et al., 2020; Karyotaki et al., 2020). Academic stress can reduce motivation, hinder academic achievement, and lead to increased college dropout rates (Pascoe et al., 2020).

Academic stress has also been shown to negatively impact mental health in students (Li and Lin, 2003; Eisenberg et al., 2009; Green et al., 2021). Mental, or psychological, well-being is one of the components of positive mental health, and it includes happiness, life satisfaction, stress management, and psychological functioning (Ryan and Deci, 2001; Tennant et al., 2007; Galderisi et al., 2015; Trout and Alsandor, 2020; Defeyter et al., 2021; Green et al., 2021). Positive mental health is an understudied but important area that helps paint a more comprehensive picture of overall mental health (Tennant et al., 2007; Margraf et al., 2020). Moreover, positive mental health has been shown to be predictive of both negative and positive mental health indicators over time (Margraf et al., 2020). Further exploring the relationship between academic stress and mental well-being is important because poor mental well-being has been shown to affect academic performance in college (Tennant et al., 2007; Eisenberg et al., 2009; Freire et al., 2016).

Perception of academic stress varies among different groups of college students (Lee et al., 2021). For instance, female college students report experiencing increased stress than their male counterparts (Misra et al., 2000; Eisenberg et al., 2007; Evans et al., 2018; Lee et al., 2021). Male and female students also respond differently to stressors (Misra et al., 2000; Verma et al., 2011). Moreover, compared to their cisgender peers, non-binary students report increased stressors and mental health issues (Budge et al., 2020). The academic year of study of the college students has also been shown to impact academic stress levels (Misra and McKean, 2000; Elias et al., 2011; Wyatt et al., 2017; Liu, C. H., et al., 2019; Defeyter et al., 2021). While several studies indicate that racial/ethnic minority groups of students, including Black/African American, Hispanic/Latino, and Asian American students, are more likely to experience anxiety, depression, and suicidality than their white peers (Lesure-Lester and King, 2004; Lipson et al., 2018; Liu, C. H., et al., 2019; Kodish et al., 2022), these studies are limited and often report mixed or inconclusive findings (Liu, C. H., et al., 2019; Kodish et al., 2022). Therefore, more studies should be conducted to address this gap in research to help identify subgroups that may be disproportionately impacted by academic stress and lower well-being.

The coronavirus disease 19 (COVID-19) pandemic is a major stressor that has led to a mental health crisis (American Psychological Association, 2020; Dong and Bouey, 2020). For college students, the COVID-19 pandemic has resulted in significant changes and disruptions to daily life, elevated stress levels, and mental and physical health deterioration (American Psychological Association, 2020; Husky et al., 2020; Patsali et al., 2020; Son et al., 2020; Clabaugh et al., 2021; Lee et al., 2021; Lopes and Nihei, 2021; Yang et al., 2021). While any college student is vulnerable to these stressors, these concerns are amplified for members of minority groups (Salerno et al., 2020; Clabaugh et al., 2021; McQuaid et al., 2021; Prowse et al., 2021; Kodish et al., 2022). Identifying students at greatest risk provides opportunities to offer support, resources, and mental health services to specific subgroups.

The overall aim of this study was to assess academic stress and mental well-being in a sample of college students. Within this umbrella, we had several goals. First, to determine whether a relationship exists between the two constructs of perceived academic stress, measured by the Perception of Academic Stress Scale (PAS), and mental well-being, measured by the Short Warwick-Edinburgh Mental Well-Being Scale (SWEMWBS), in college students. Second, to identify groups that could experience differential levels of academic stress and mental health. Third, to explore how the perception of the ongoing COVID-19 pandemic affected stress levels. We hypothesized that students who experienced more academic stress would have worse psychological well-being and that certain groups of students would be more impacted by academic- and COVID-19-related stress.

## Materials and Methods

### Survey Instrument

A survey was developed that included all questions from the Short Warwick-Edinburgh Mental Well-Being (Tennant et al., 2007; Stewart-Brown and Janmohamed, 2008) and from the Perception of Academic Stress Scale (Bedewy and Gabriel, 2015). The Short Warwick-Edinburgh Mental Well-Being Scale is a seven-item scale designed to measure mental well-being and positive mental health (Tennant et al., 2007; Fung, 2019; Shah et al., 2021). The Perception of Academic Stress Scale is an 18-item scale designed to assess sources of academic stress perceived by individuals and measures three main academic stressors: academic expectations, workload and examinations, and academic self-perceptions of students (Bedewy and Gabriel, 2015). These shorter scales were chosen to increase our response and study completion rates (Kost and de Rosa, 2018). Both tools have been shown to be valid and reliable in college students with Likert scale responses (Tennant et al., 2007; Bedewy and Gabriel, 2015; Ringdal et al., 2018; Fung, 2019; Koushede et al., 2019). Both the SWEMWBS and PAS scores are a summation of responses to the individual questions in the instruments. For the SWEMWBS questions, a higher score indicates better mental health, and scores range from 7 to 35. Similarly, the PAS questions are phrased such that a higher score indicates lower levels of stress, and scores range from 18 to 90. We augmented the survey with demographic questions (e.g., age, gender, and race/ethnicity) at the beginning of the survey and two yes/no questions and one Likert scale question about the impact of the COVID-19 pandemic at the end of our survey.

### Sample

Participants for the study were self-reported college students between the ages of 18 and 30 years who resided in the United States, were fluent in English, and had Internet access. Participants were solicited through Prolific (https://prolific.co) in October 2021. A total of 1,023 individuals enrolled in the survey. Three individuals did not agree to participate after beginning the survey. Two were not fluent in English. Thirteen individuals indicated that they were not college students. Two were not in the 18–30 age range, and one was located outside of the United States. Of the remaining individuals, 906 were full-time students and 96 were part-time students. Given the skew of the data and potential differences in these populations, we removed the part-time students. Of the 906 full-time students, 58 indicated that they were in their fifth year of college or higher. We understand that not every student completes their undergraduate studies in 4 years, but we did not want to have a mixture of undergraduate and graduate students with no way to differentiate them. Finally, one individual reported their age as a non-number, and four individuals did not answer a question about their response to the COVID-19 pandemic. This yielded a final sample of 843 college students.

### Data Analyses

After reviewing the dataset, some variables were removed from consideration due to a lack of consistency (e.g., some students reported annual income for themselves and others reported family income) or heterogeneity that prevented easy categorization (e.g., field of study). We settled on four variables of interest: gender, race/ethnicity, year in school, and response to the COVID-19 pandemic (Table 1). Gender was coded as female, male, or non-binary. Race/ethnicity was coded as white or Caucasian; Black or African American; East Asian; Hispanic, Latino, or of Spanish origin; or other. Other was used for groups that were not well-represented in the sample and included individuals who identified themselves as Middle Eastern, Native American or Alaskan Native, and South Asian, as well as individuals who chose “other” or “prefer not to answer” on the survey. The year of study was coded as one through four, and COVID-19 stress was coded as two groups, no change/neutral response/reduced stress or increased stress.

**Table 1:** Characteristics of the participants in the study.

**Gender**			**Race/ethnicity**			**Year of study**			**Response to COVID-19**		
Female	662	78.5%	White or Caucasian	560	66.4%	1	134	15.9%	No impact/ neutral response/decreased stress	165	19.6%
Male	141	16.7%	Black or African American	66	7.8%	2	233	27.6%			
Nonbinary	40	4.7%	East Asian	78	9.3%	3	251	29.8%	Increased stress	678	80.4%
			Hispanic, Latino, or of Spanish origin	74	8.8%	4	225	26.7%			
			Other	65	7.7%						

Our first goal was to determine whether there was a relationship between self-reported academic stress and mental health, and we found a significant correlation (see Results section). Given the positive correlation, a multivariate analysis of variance (MANOVA) with a model testing the main effects of gender, race/ethnicity, and year of study was run in SPSS v 26.0. A factorial MANOVA would have been ideal, but our data were drawn from a convenience sample, which did not give equal representation to all groupings, and some combinations of gender, race/ethnicity, and year of study were poorly represented (e.g., a single individual). As such, we determined that it would be better to have a lack of interaction terms as a limitation to the study than to provide potentially spurious results. Finally, we used chi-square analyses to assess the effect of potential differences in the perception of the COVID-19 pandemic on stress levels in general among the groups in each category (gender, race/ethnicity, and year of study).

## Results

In terms of internal consistency, Cronbach's alpha was 0.82 for the SMEMWBS and 0.86 for the PAS. A variety of descriptors have been applied to Cronbach's alpha values. That said, 0.7 is often considered a threshold value in terms of acceptable internal consistency, and our values could be considered “high” or “good” (Taber, 2018).

The participants in our study were primarily women (78.5% of respondents; Table 1). Participants were not equally distributed among races/ethnicities, with the majority of students selecting white or Caucasian (66.4% of responders; Table 1), or years of study, with fewer first-year students than other groups (Table 1).

**Figure 1:**
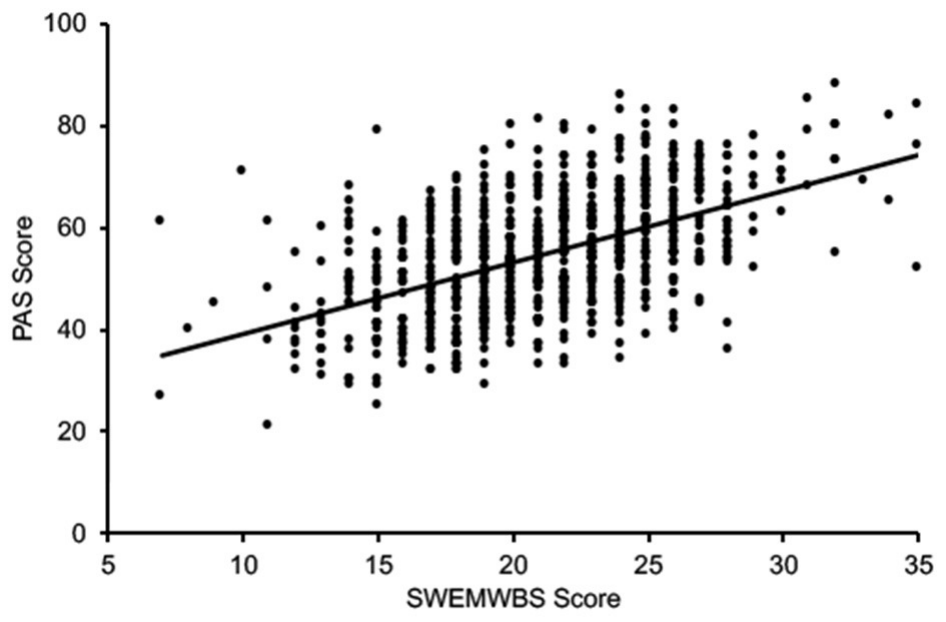
SWEMWBS and PAS scores for all participants.

Students who reported higher academic stress also reported worse mental well-being in general, irrespective of age, gender, race/ethnicity, or year of study. PAS and SWEMWBS scores were significantly correlated (*r* = 0.53, *p* < 0.001; Figure 1), indicating that a higher level of perceived academic stress is associated with worse mental well-being in college students within the United States.

**Figure 2:**
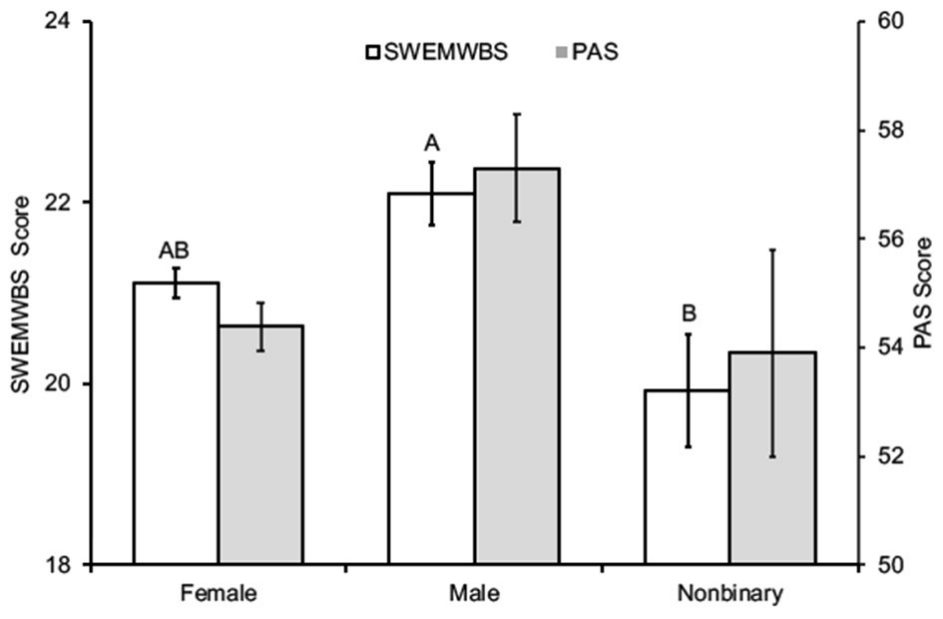
SWEMWBS and PAS scores according to gender (mean ± SEM). Different letters for SWEMWBS scores indicate different statistical groupings (*p* < 0.05).

**Figure 3:**
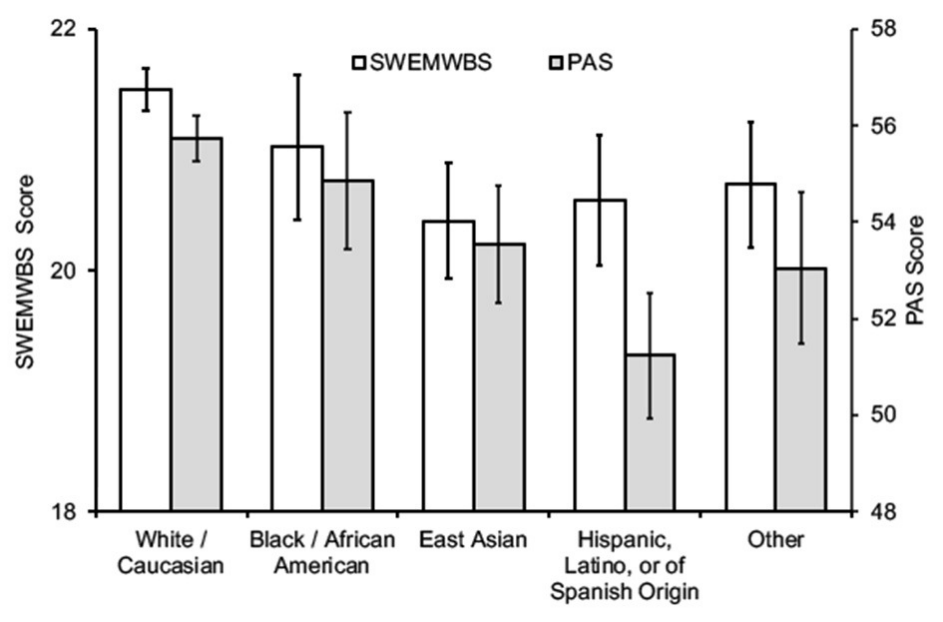
SWEMWBS and PAS scores according to race/ethnicity (mean ± SEM).

**Figure 4:**
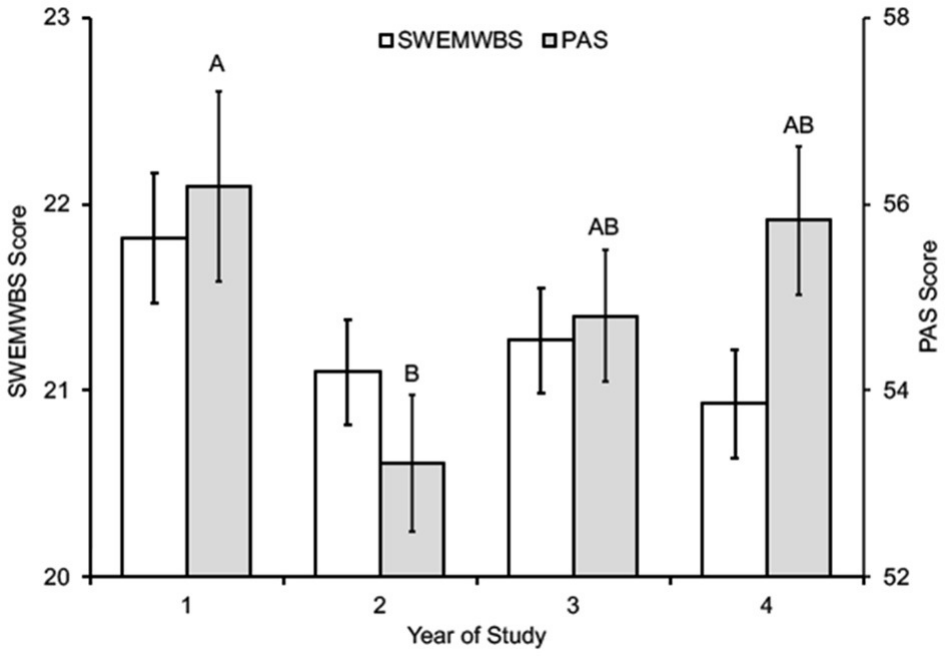
SWEMWBS and PAS scores according to year in college (mean ± SEM). Different letters for PAS scores indicate different statistical groupings (*p* < 0.05).

Among the subgroups of students, women, non-binary students, and second-year students reported higher academic stress levels and worse mental well-being (Table 2; Figures 2–4). In addition, the combined measures differed significantly between the groups in each category (Table 2). However, as measured by partial eta squared, the effect sizes were relatively small, given the convention of 0.01 = small, 0.06 = medium, and 0.14 = large differences (Lakens, 2013). As such, there were only two instances in which Tukey's *post-hoc* tests revealed more than one statistical grouping (Figures 2–4). For SWEMWBS score by gender, women were intermediate between men (high) and non-binary individuals (low) and not significantly different from either group (Figure 2). Second-year students had the lowest PAS scores for the year of study, and first-year students had the highest scores. Third- and fourth-year students were intermediate and not statistically different from the other two groups (Figure 4). There were no pairwise differences in academic stress levels or mental well-being among racial/ethnic groups.

**Table 2:** Results of the MANOVA.

	**Pillai's trace**	***F***	***p***	**Partial eta squared**
Gender	0.018	3.86	0.004	0.009
Race/ethnicity	0.022	2.32	0.02	0.011
Year of study	0.016	2.24	0.04	0.008

The findings varied among categories in terms of stress responses due to the COVID-19 pandemic (Table 3). For gender, men were less likely than women or non-binary individuals to report increased stress from COVID-19 (χ^2^= 27.98, df = 2, *p* < 0.001). All racial/ethnic groups responded similarly to the pandemic (χ^2^= 3.41, df = 4, *p* < 0.49). For the year of study, first-year students were less likely than other cohorts to report increased stress from COVID-19 (χ^2^= 9.38, df = 3, *p* < 0.03).

**Table 3:** Impact of COVID-19 on stress level by gender, race/ethnicity, and year of study.

	**No change/neutral**		**Increased stress**	
	**response/ reduced stress**			
	***n***	**%**	***n***	**%**
**Gender**				
Female	118	17.8	544	82.2
Male	47	33.3	94	66.7
Nonbinary	0	0	40	100
**Race/ethnicity**				
White or Caucasian	104	18.6	456	81.4
Black or African American	16	24.2	50	75.8
East Asian	20	25.6	58	74.4
Hispanic, Latino, or of Spanish origin	14	18.9	60	81.1
Middle Eastern, Native American, Alaskan Native, South Asian, other, or prefer not to answer	11	16.9	54	83.1
**Year of study**				
1	38	28.4	96	71.6
2	43	18.5	190	81.5
3	39	15.5	212	84.5
4	45	20	180	80

## Discussion

Our primary findings showed a positive correlation between perceived academic stress and mental well-being in United States college students, suggesting that academic stressors, including academic expectations, workload and grading, and students' academic self-perceptions, are equally important as psychological well-being. Overall, irrespective of gender, race/ethnicity, or year of study, students who reported higher academic stress levels experienced diminished mental well-being. The utilization of well-established scales and a large sample size are strengths of this study. Our results extend and contribute to the existing literature on stress by confirming findings from past studies that reported higher academic stress and lower psychological well-being in college students utilizing the same two scales (Green et al., 2021; Syed, 2021). To our knowledge, the majority of other prior studies with similar findings examined different components of stress, studied negative mental health indicators, used different scales or methods, employed smaller sample sizes, or were conducted in different countries (Li and Lin, 2003; American Psychological Association, 2020; Husky et al., 2020; Pascoe et al., 2020; Patsali et al., 2020; Clabaugh et al., 2021; Lee et al., 2021; Lopes and Nihei, 2021; Yang et al., 2021).

This study also demonstrated that college students are not uniformly impacted by academic stress or pandemic-related stress and that there are significant group-level differences in mental well-being. Specifically, non-binary individuals and second-year students were disproportionately impacted by academic stress. When considering the effects of gender, non-binary students, in comparison to gender-conforming students, reported the highest stress levels and worst psychological well-being. Although there is a paucity of research examining the impact of academic stress in non-binary college students, prior studies have indicated that non-binary adults face adverse mental health outcomes when compared to male and female-identifying individuals (Thorne et al., 2018; Jones et al., 2019; Budge et al., 2020). Alarmingly, Lipson et al. (2019) found that gender non-conforming college students were two to four times more likely to experience mental health struggles than cisgender students (Lipson et al., 2019). With a growing number of college students in the United States identifying as as non-binary, additional studies could offer invaluable insight into how academic stress affects this population (Budge et al., 2020).

In addition, we found that second-year students reported the most academic-related distress and lowest psychological well-being relative to students in other years of study. We surmise this may be due to this group taking advanced courses, managing heavier academic workloads, and exploring different majors. Other studies support our findings and suggest higher stress levels could be attributed to increased studying and difficulties with time management, as well as having less well-established social support networks and coping mechanisms compared to upperclassmen (Allen and Hiebert, 1991; Misra and McKean, 2000; Liu, X et al., 2019). Benefiting from their additional experience, upperclassmen may have developed more sophisticated studying skills, formed peer support groups, and identified approaches to better manage their academic stress (Allen and Hiebert, 1991; Misra and McKean, 2000). Our findings suggest that colleges should consider offering tailored mental health resources, such as time management and study skill workshops, based on the year of study to improve students' stress levels and psychological well-being (Liu, X et al., 2019).

Although this study reported no significant differences regarding race or ethnicity, this does not indicate that minority groups experienced less academic stress or better mental well-being (Lee et al., 2021). Instead, our results may reflect the low sample size of non-white races/ethnicities, which may not have given enough statistical power to corroborate. In addition, since coping and resilience are important mediators of subjective stress experiences (Freire et al., 2020), we speculate that the lower ratios of stress reported in non-white participants in our study (75 vs. 81) may be because they are more accustomed to adversity and thereby more resilient (Brown, 2008; Acheampong et al., 2019). Furthermore, ethnic minority students may face stigma when reporting mental health struggles (Liu, C. H., et al., 2019; Lee et al., 2021). For instance, studies showed that Black/African American, Hispanic/Latino, and Asian American students disclose fewer mental health issues than white students (Liu, C. H., et al., 2019; Lee et al., 2021). Moreover, the ability to identify stressors and mental health problems may manifest differently culturally for some minority groups (Huang and Zane, 2016; Liu, C. H., et al., 2019). Contrary to our findings, other studies cited racial disparities in academic stress levels and mental well-being of students. More specifically, Negga et al. (2007) concluded that African American college students were more susceptible to higher academic stress levels than their white classmates (Negga et al., 2007). Another study reported that minority students experienced greater distress and worse mental health outcomes compared to non-minority students (Smith et al., 2014). Since there may be racial disparities in access to mental health services at the college level, universities, professors, and counselors should offer additional resources to support these students while closely monitoring their psychological well-being (Lipson et al., 2018; Liu, C. H., et al., 2019).

While the COVID-19 pandemic increased stress levels in all the students included in our study, women, non-binary students, and upperclassmen were disproportionately affected. An overwhelming body of evidence suggests that the majority of college students experienced increased stress levels and worsening mental health as a result of the pandemic (Allen and Hiebert, 1991; American Psychological Association, 2020; Husky et al., 2020; Patsali et al., 2020; Son et al., 2020; Clabaugh et al., 2021; Lee et al., 2021; Yang et al., 2021). Our results also align with prior studies that found similar subgroups of students experience disproportionate pandemic-related distress (Gao et al., 2020; Clabaugh et al., 2021; Hunt et al., 2021; Jarrett et al., 2021; Lee et al., 2021; Chen and Lucock, 2022). In particular, the differences between female students and their male peers may be the result of different psychological and physiological responses to stress reactivity, which in turn may contribute to different coping mechanisms to stress and the higher rates of stress-related disorders experienced by women (Misra et al., 2000; Kajantie and Phillips, 2006; Verma et al., 2011; Gao et al., 2020; Graves et al., 2021). COVID-19 was a secondary consideration in our study and survey design, so the conclusions drawn here are necessarily limited.

The implications of this study are that college students facing increased stress and struggling with mental health issues should receive personalized and specific mental health services, resources, and support. This is particularly true for groups that have been disproportionately impacted by academic stress and stress due to the pandemic. Many students who experience mental health struggles underutilize college services due to cost, stigma, or lack of information (Cage et al., 2020; Lee et al., 2021). To raise awareness and destigmatize mental health, colleges can consider distributing confidential validated assessments, such as the PAS and SWEMWBS, in class and teach students to self-score (Lee et al., 2021). These results can be used to understand how academic stress and mental well-being change over time and allow for specific and targeted interventions for vulnerable groups. In addition, teaching students healthy stress management techniques has been shown to improve psychological well-being (Alborzkouh et al., 2015). Moreover, adaptive coping strategies, including social and emotional support, have been found to improve the mental well-being of students, and stress-reduction peer support groups and workshops on campus could be beneficial in reducing stress and improving the self-efficacy of students (Ruthig et al., 2009; Baqutayan, 2011; Bedewy and Gabriel, 2015; Freire et al., 2020; Green et al., 2021; Suresh et al., 2021). Other interventions that have been effective in improving the coping skills of college students include cognitive-behavioral therapy, mindfulness mediation, and online coping tools (Kang et al., 2009; Regehr et al., 2013; Molla Jafar et al., 2015; Phang et al., 2015; Houston et al., 2017; Yusufov et al., 2019; Freire et al., 2020). Given that resilience has also been shown to help mediate stress and improve mental well-being during the COVID-19 pandemic, interventions focusing on enhancing resilience should be considered (Surzykiewicz et al., 2021; Skalski et al., 2022). Telemental health resources across colleges can also be implemented to reduce stigma and improve at-risk students' access to care (Toscos et al., 2018; Hadler et al., 2021). University campuses, professors, and counselors should consider focusing on fostering a more equitable and inclusive environment to encourage marginalized students to seek mental health support (Budge et al., 2020).

## Limitations

While our study has numerous strengths, including using standardized instruments and a large sample size, this study also has several limitations due to both the methodology and sample. First, the correlational study design precludes making any causal relationships (Misra and McKean, 2000). Thereby, our findings should be taken in the context of academic stress and mental well-being, and recognize that mental health could be caused by other non-academic factors. Second, the PAS comprised only the perception of responses to academic stress, but stress is a multi-factorial response that encompasses both perceptions and coping mechanisms to different stressors, and the magnitude of stress varies with the perception of the degree of uncontrollability, unpredictability, or threat to self (Miller, 1981; Hobfoll and Walfisch, 1984; Lazarus and Folkman, 1984; Wheaton, 1985; Perrewé and Zellars, 1999; Schneiderman et al., 2005; Bedewy and Gabriel, 2015; Schönfeld et al., 2016; Reddy et al., 2018; Freire et al., 2020; Karyotaki et al., 2020). Third, the SWEMSBS used in our study and the data only measured positive mental health. Mental health pathways are numerous and complex, and are composed of distinct and interdependent negative and positive indicators that should be considered together (Margraf et al., 2020). Fourth, due to the small effect sizes and unequal representation for different combinations of variables, our analysis for both the PAS and SWEMSBS included only summed-up scales and did not examine group differences in response to the type of academic stressors or individual mental health questions.

An additional limitation is that the participants in our study were a convenience sample. The testing service we used, prolific.co, self-reports a sample bias toward young women of high levels of education (i.e., WEIRD bias) (Team Prolific, 2018). The skew toward this population was observed in our data, as 80% of our participants were women. While we controlled for these factors, the possibility remains that the conclusions we draw for certain groups, such as nonbinary students, ethnic/racial minorities, and men, may not be as statistically powerful as they should be. Moreover, our pre-screening was designed to recruit undergraduate level, English-speaking, 18–30-year-olds who resided in the United States. This resulted in our participant demographics being skewed toward the WEIRD bias that was already inherent in the testing service we used. Future research will aim to be more inclusive of diverse races/ethnicities, sexual orientations, languages, educational backgrounds, socioeconomic backgrounds, and first-generation college students.

Another limitation of our study is the nature of satisficing. Satisficing is a response strategy in which a participant answers a question to satisfy its condition with little regard to the quality or accuracy of the answer (Roberts et al., 2019). Anonymous participants are more likely to satisfice than respondents who answer the question face-to-face (Krosnick et al., 2002). We sought to mitigate satisficing by offering financial incentives to increase response rates and decrease straight-lining, item skipping, total missing items, and non-completion (Cole et al., 2015). Concerns of poor data quality due to surveys offering financial incentives found little evidence to support that claim and may do the opposite (Cole et al., 2015). On the other hand, social desirability bias may have influenced the participant's self-reported responses, although our anonymous survey design aimed to reduce this bias (Joinson, 1999; Kecojevic et al., 2020).

## Future Studies

Future studies should replicate our study to validate our results, conduct longitudinal cohort studies to examine well-being and perceived academic stress over time, and aim for a more representative student sample that includes various groups, including diverse races/ethnicities, sexual orientations, socioeconomic backgrounds, languages, educational levels, and first-generation college students. Additionally, these studies should consider examining other non-academic stressors and students' coping mechanisms, both of which contribute to mental health and well-being (Lazarus and Folkman, 1984; Freire et al., 2020). Further explorations of negative and other positive indicators of mental health may offer a broader perspective (Margraf et al., 2020). Moreover, future research should consider extending our work by exploring group differences in relation to each factor in the PAS (i.e., academic expectations, workload and examinations, and self-perception of students) and SWEMBS to determine which aspects of academic stress and mental health were most affected and allow for the devising of targeted stress-reduction approaches. Ultimately, we hope our research spurs readers into advocating for greater academic support and access to group-specific mental health resources to reduce the stress levels of college students and improve their mental well-being.

## Conclusion

Utilizing two well-established scales, our research found a statistically significant correlation between the perceived academic stress of university students and their mental well-being (i.e., the higher the stress, the worse the well-being). This relationship was most apparent among gender and grade levels. More specifically, non-binary and second-year students experienced greater academic burden and lower psychological well-being. Moreover, women, non-binary students, and upper-level students were disproportionately impacted by stress related to the COVID-19 pandemic.

Studies regarding broad concepts of stress and well-being using a questionnaire are limited, but our study adds value to the understanding of academic stress as a contributor to the overall well-being of college students during this specific point in time (i.e., the COVID-19 pandemic). Competition both for admission to college (Bound et al., 2009) and during college (Posselt and Lipson, 2016) has increased over time. Further, selective American colleges and universities draw applicants from a global pool. As such, it is important to document the dynamics of academic stress with renewed focus. We hope that our study sparks interest in both exploring and funding in-depth and well-designed psychological studies related to stress in colleges in the future.

## Data Availability Statement

The raw data supporting the conclusions of this article will be made available by the authors, without undue reservation.

## Ethics Statement

The studies involving human participants were reviewed and approved by Institutional Review Board at Rutgers University. The patients/participants provided their written informed consent to participate in this study.

## Author Contributions

GB and MB contributed to conceptualization, study design, IRB application, manuscript drafting, and revision. XZ participated in the conceptualization and design of the questionnaires. HB participated in subject recruitment and questionnaire collection. KP contributed to data analysis, table and figure preparation, manuscript drafting, and revision. XM contributed to conceptualization, study design, IRB application, supervision of the project, manuscript drafting, and revision. All authors contributed to the article and approved the submitted version.

## Funding

This study was made possible by a generous donation from the Knights of Columbus East Hanover Chapter in New Jersey.

## Conflict of Interest

The authors declare that the research was conducted in the absence of any commercial or financial relationships that could be construed as a potential conflict of interest.

## Publisher's Note

All claims expressed in this article are solely those of the authors and do not necessarily represent those of their affiliated organizations, or those of the publisher, the editors and the reviewers. Any product that may be evaluated in this article, or claim that may be made by its manufacturer, is not guaranteed or endorsed by the publisher.
